# Nintedanib administration after the onset of acute exacerbation of interstitial lung disease in the real world

**DOI:** 10.1038/s41598-023-39101-w

**Published:** 2023-08-02

**Authors:** Motoyasu Kato, Shinichi Sasaki, Wataru Mori, Makiko Kohmaru, Takashi Akimoto, Eri Hayakawa, Soichiro Soma, Yuta Arai, Naho Sakamoto Matsubara, Shun Nakazawa, Takuto Sueyasu, Haruki Hirakawa, Hiroaki Motomura, Issei Sumiyoshi, Yusuke Ochi, Junko Watanabe, Kazuaki Hoshi, Kotaro Kadoya, Hiroaki Ihara, Jia Hou, Shinsaku Togo, Kazuhisa Takahashi

**Affiliations:** Department of Respiratory Medicine, Graduate School of Medicine, Juntendo University, 3-1-3 Hongo, Bunkyo-ku, Tokyo, 113-8431 Japan

**Keywords:** Respiratory tract diseases, Outcomes research

## Abstract

Nintedanib reduces the decline in forced vital capacity and extends the time to the first acute exacerbation of interstitial lung disease (AE-ILD). However, the effect of additional nintedanib administration after AE-ILD onset is unknown. This study aimed to investigate the efficacy and safety of nintedanib administration after AE-ILD development. We retrospectively collected the data of 33 patients who developed AE-ILD between April 2014 and January 2022. Eleven patients who received nintedanib after AE-ILD development and the remaining who did not were classified into the N and No-N groups, respectively. The survival time in the N group tended to be longer than that in the No-N group. The generalized Wilcoxson test revealed that the cumulative mortality at 90 days from AE-ILD onset was significantly lower in the N group. The time to subsequent AE-ILD development was significantly longer in the N group than that in the No-N group. The incidence of adverse gastrointestinal effects and liver dysfunction in the N group was 9–18%. Treatment without nintedanib after AE-ILD development and the ratio of arterial oxygen partial pressure to fractional inspired oxygen were significant independent prognostic factors in the multivariate analysis. Thus, nintedanib administration may be a treatment option for AE-ILD.

## Introduction

Acute exacerbation of interstitial lung disease (AE-ILD) is a severe disease characterized by acute progressive respiratory failure^1–6^, with an incidence of approximately 10% in Japanese patients with idiopathic pulmonary fibrosis (IPF)^7,8^. Chronic fibrosing interstitial lung diseases (ILDs) include IPF, idiopathic non-specific interstitial pneumonia (NSIP), fibrotic hypersensitivity pneumonia (HP), and connective tissue disease-associated interstitial lung disease (CTD-ILD)^9,10^. Despite empirical treatment with corticosteroids (steroid pulse therapy), patients who develop AE-ILD show a very high 1-month mortality rate (30–50%)^2^. Several clinical trials focused on AE-ILD, particularly the acute exacerbation of IPF (AE-IPF), have been conducted using several new medications, including intravenous cyclophosphamide pulse therapy and recombinant human soluble thrombomodulin; however, most trials have shown that these medications are not effective for the treatment of AE-IPF^11,12^. Therefore, very few options, including corticosteroids, are currently available for the treatment of AE-ILD.

Nintedanib is an anti-fibrotic agent for IPF, systemic sclerosis-induced interstitial lung disease (SSc-ILD), and progressive fibrosing interstitial lung disease (PF-ILD) that inhibits the vascular endothelial growth factor (VEGF)-2, platelet-derived growth factor, and fibroblast growth factor receptors^13,14^. In the INPULSIS trial for IPF, SCENCIS trial for SSc-ILD, and INBUILD trial for PF-ILD, nintedanib inhibited the chronic decline in forced vital capacity (FVC) and extended the time to the initial development of AE-ILD^15–18^. Moreover, in the Japanese population of the INPULSIS cohort, the incidence of AE was lower in patients who received nintedanib than that in those who received a placebo (4% vs. 12%)^8^.

VEGF is associated with the pathogenesis of AE-ILD and acute respiratory distress syndrome (ARDS)^19^. In particular, the binding of VEGF to VEGF receptor-2 is associated with vascular hyperpermeability through Src kinase activation^20^. The pathological findings of AE-ILD include diffuse alveolar damage, similar to that caused by ARDS^21^. Therefore, prior treatment with nintedanib, which suppresses VEGF receptor expression, may inhibit the development of AE-ILD. Anti-VEGF receptor inhibitors and antibodies are usually used as anti-cancer agents, particularly in patients with advanced lung cancer. The incidence of drug-induced interstitial lung disease was significantly lower in the patients who received chemotherapy with a VEGF inhibitor than that in those who received chemotherapy without a VEGF inhibitor^22–24^.

In Japan, nintedanib was initiated for AE-ILD in two IPF cases. Ito et al. described a case of AE-IPF that was treated with nintedanib alone^25^. Tomioka et al. also reported another case in which only nintedanib was effective in treating AE-IPF^26^. However, no previous studies, particularly cohort studies, have evaluated the effect of nintedanib on the AE-IPF period, and no description of anti-fibrotic agents for AE-IPF or AE-ILD has been provided in the 2017 Japanese guidelines for the treatment of IPF^27^ or the 2018 and 2022 IPF international guidelines^9,28–30^. Therefore, we hypothesized that nintedanib administration after the development of AE-ILD is a new treatment option for AE-ILD and performed this study to investigate the efficacy and safety of nintedanib initiation in the AE-ILD phase using our retrospective cohort.

## Results

### Patient background

**Figure 1:**
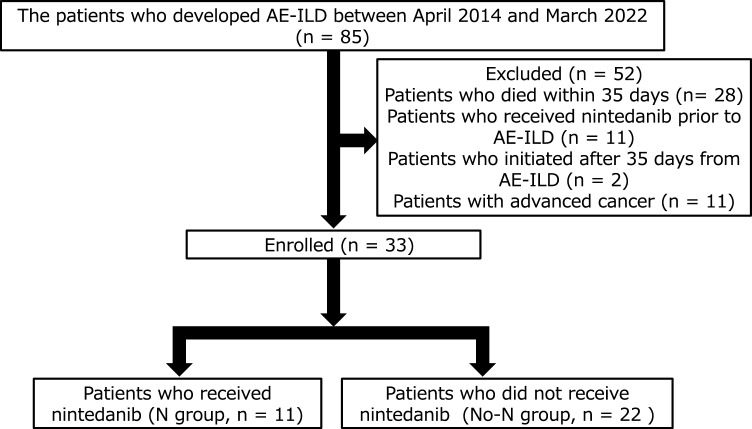
Study patients. *AE-ILD* acute exacerbation of interstitial lung disease.

Among the 33 enrolled patients who developed AE-ILD, 11 started treatment with nintedanib between three and 35 days after the development of AE-ILD and were classified into the N group. The remaining 22 patients who did not receive nintedanib after the development of AE-ILD were categorized into the No-N group (Fig. 1). The N group included five patients who had experienced AE-ILD episodes previously, while the No-N group included no patient with a history of AE-ILD development. The patient background characteristics are presented in Table 1. Although the median age was significantly higher in the N group, the other patient characteristics, including sex, smoking history, baseline pulmonary function, diagnosis of baseline ILD, and treatment for baseline ILD, did not differ significantly between the groups. Ten patients in the N group and 14 in the No-N group were diagnosed with IPF prior to the onset of AE-ILD. Among the patients with ILD other than IPF, three had rheumatoid arthritis (RA)-induced ILD, two had vasculitis, one had idiopathic fibrotic NSIP, and two had fibrotic HP in the No-N group. One patient in the N group was diagnosed with fibrotic idiopathic NSIP. No patients had reversible pathogenesis such as cryptogenic organizing pneumonia in either group. Although one patient was diagnosed with fibrotic idiopathic NSIP, lung fibrosis was slowly progressive. The patients met the criteria for PF-ILD^31^. The HRCT findings showed progressive lung fibrosis, including honeycomb and severe traction bronchiectasis. Therefore, all patients in the N group were diagnosed with IPF or PF-ILD, for which the administration of nintedanib is approved. Three patients with RA-ILD and two with vasculitis showed a usual interstitial pneumonia pattern (UIP) pattern or probable UIP pattern on HRCT. The other two patients with idiopathic fibrotic NSIP had an alternative diagnosis pattern (fibrotic NSIP pattern) on HRCT in the No-N group. The two groups showed no significant differences in the frequency of IPF and HRCT patterns. A few patients in each group received pirfenidone at an effective therapeutic dose. The frequency of the patients who received pirfenidone did not differ significantly between the groups. A few other patients received steroids for baseline idiopathic fibrotic NSIP before the development of AE-ILD. During the assessments of pulmonary function, no significant differences were observed in FVC and diffusion capacity between the two groups.

**Table 1:** Patient background.

Characteristics	Total patients n = 33	N group n = 11	No-N group n = 22	*p*
Age (years)	75.91 ± 8.72	80.54 ± 5.76	73.59 ± 9.11	0.045
Sex				0.228
Female/male	7/26	1/10	6/16	
Smoking history				0.097
No/yes	9/24	1/10	8/14	
Baseline ILD				0.097
No IPF/IPF	9/24	1/10	8/14	
Baseline HRCT pattern				
UIP + probable				1.000
UIP/fibrotic NSIP	30/3	10/1	20/2	
Prior treatment (pirfenidone)				0.376
No/yes	28/5	8/3	20/2	
Prior treatment (prednisolone)				0.798
No/yes	29/4	10/1	19/3	
%FVC (%)	71.15 ± 19.54 n = 24	76.02 ± 16.46 n = 10	67.68 ± 21.37 n = 14	0.313
%DLco (%)	33.44 ± 13.58 n = 19	30.78 ± 6.97 n = 9	35.84 ± 17.64 n = 10	0.434
Serum markers at the diagnosis of AE-IPF				
P/F ratio	239.41 ± 102.75	236.26 ± 31.4710.30	240.98 ± 22.25	0.903
WBC (/µL)	11,241.29 ± 3528.61	10,834.50 ± 4366.50	11,465.00 ± 799.59	0.642
CRP (mg/dL)	9.32 ± 7.33	6.89 ± 6.30	10.65 ± 7.66	0.176
D-dimer (µg/mL)	7.77 ± 11.65	5.80 ± 4.51	8.81 ± 14.06	0.517
LDH (U/L)	360 ± 896.97	310 ± 61.56	387 ± 103.21	0.032
KL-6 (U/L)	1339.61 ± 786.31 n = 31	1253.09 ± 240.32 n = 11	1387.20 ± 916.61 n = 20	0.657
SP-D (mg/dL)	491.01 ± 370.36 n = 29	516.17 ± 436.08 n = 10	475.64 ± 336.75 n = 19	0.780

*ILD* interstitial lung disease, *IPF* idiopathic pulmonary fibrosis, *UIP* usual interstitial pneumonia, *HRCT* high resolution computed tomography, *NSIP* non-specific interstitial pneumonia, *AE* acute exacerbation of idiopathic pulmonary fibrosis, *FVC* forced vital capacity, *DLco* diffusing capacity for carbon monoxide, *P/F ratio* PaO_2_/FiO_2_ ratio, *WBC* white blood cell, *CRP* c-related peptide, *LDH* lactate dehydrogenase, *KL-6* Krebs von den Lungen-6, *SP-D* surfactant protein-D.

We evaluated the intergroup differences in the serum markers associated with AE-ILD. At the onset of AE-ILD, although the median serum lactate dehydrogenase level was significantly lower in the N group than that in the No-N group, the serum markers associated with AE-ILD, including the Krebs von den Lungen-6, surfactant protein D, white blood cell count, and C-reactive protein levels, did not differ significantly between the groups. The P/F ratio at the onset of AE-ILD also did not differ significantly between the groups. Thus, the severity of AE-ILD at onset was roughly equivalent between the two groups. The two groups showed no significant differences in pulmonary function, including FVC and diffusion capacity.

All patients received treatments other than nintedanib for AE-ILD. All patients received corticosteroids, including steroid pulse therapy and antibiotics. The frequency of intravenous cyclophosphamide pulse therapy tended to be higher in the No-N group than that in the N group. In contrast, the two groups showed no significant differences in the frequencies of other treatments for AE-ILD (Table 2).

**Table 2:** Other treatment for AE-ILD.

Treatment	Total patients n = 33	N group n = 11	No-N group n = 22	*p*
Steroid pulse				1.000
No/yes	0/33	0/11	0/22	
IVCY				0.026
No/yes	15/18	8/3	7/15	
Immunosuppressant				0.046
No/yes	19/14	9/2	10/12	
Antibiotics				1.000
No/yes	0/33	0/11	0/22	

*AE-ILD* acute exacerbation of interstitial lung disease, *IVCY* intravenous cyclophosphamide.

### Survival and time to subsequent AE-ILD

**Figure 2:**
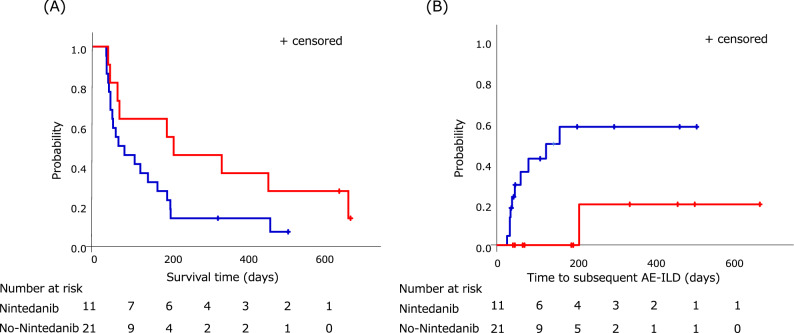
Kaplan–Meier curve for survival time and the time to subsequent AE-ILD. (**A**) The red line shows the survival for patients in the N group. The blue line shows the survival in the No-N group. The median survival time in the N group tended to be longer than that in the No-N group. (**B**) The red line shows the time to the subsequent AE-ILD for patients in the N group. The blue line shows the time to the subsequent AE-ILD in the No-N group. The median survival time in the N group was significantly longer than that in the No-N group.

We evaluated the survival time from the onset of AE-ILD. The median survival time (MST) tended to be longer in the N group (213 days [95% CI 148.630–504.315 days] in the N group [red line] vs. 75 days [95% CI 33.419–132.502 days] in the No-N group [blue line]; HR 0.304; p = 0.071; log-rank test; HR 0.3497, p = 0.090; Wilcoxon test, Fig. 2A). Moreover, the generalized Wilcoxon test revealed that the cumulative mortality rate at 90 days was significantly lower in the N group than that in the No-N group (36.36% in the N group, 54.55% in the No-N group, and 48.48% in the total patients [HR 0.3061, p = 0.070; log-rank test, HR 0.2256, p = 0.048; Wilcoxon test]).

We also analyzed the difference in the time to the subsequent AE-ILD from the onset of AE-ILD. Although the incidence of subsequent AE-ILD did not reach 50%, the median time to the subsequent AE-ILD was significantly longer in the N group than that in the No-N group (MST: not reached [NR]; 95% CI NR-NR in the N group [red line] vs. MST: NR; 95% CI NR-NR in the No-N group [blue line]; HR 0.181, p = 0.019; log-rank test, HR 0.1742, p = 0.016; Wilcoxon test; Fig. 2B).

Nine patients died in the N group. Five patients died of chronic respiratory failure, two died of bacterial pneumonia, and two died of heart failure.

### Safety

Diarrhea, nausea/anorexia, and liver dysfunction are the major adverse effects of nintedanib. We evaluated the incidence of adverse effects of following nintedanib treatment in the N group using the Common Terminology Criteria for Adverse Events (CTCAE) Ver. 5.0. None of the patients reported severe adverse effects associated with nintedanib treatment, and no patient died of nintedanib-related adverse effects (CTCAE grade over 3). The incidence rates of diarrhea, nausea/anorexia, and liver dysfunction were 9.09%, 9.09%, and 18.18%, respectively, in the N group. These rates were lower than those in the chronic phase of IPF or PF-ILD in the INPULSIS and INBUILD trials^15,18^. The initial dose of nintedanib was 300 mg/day (normal dose) in eight of the 11 patients. The remaining patients received a low dose of nintedanib (200 mg/day) from the initiation of treatment due to poor general condition, such as low PS or old age. We tapered the nintedanib dose to 200 mg/day in four of the eight patients with adverse effects.

### Risk factors associated with early death within 90 days of developing AE-ILD

We performed univariate and multivariate analyses to evaluate the risk factors associated with early death (< 90 days) after the development of AE-ILD. Fourteen patients died within 90 days after the development of AE-ILD. In the univariate analysis, the P/F ratio at the onset of AE-ILD and treatment without nintedanib were significantly associated with early death (Table 3). In multivariate analysis, both P/F ratio and treatment without nintedanib were independent risk factors significantly related to early death within 90 days after the development of AE-ILD (odds ratio = 0.989, 95% CI 0.974–0.997, and p = 0.009 for the P/F ratio; odds ratio = 0.107, 95% CI 0.009–0.696, and p = 0.016 for nintedanib treatment; Table 4). Therefore, nintedanib initiation after the development of AE-ILD may be effective for improving the prognosis of AE-ILD. We also evaluated the risk factors for AE-ILD relapse after the first AE-ILD onset. However, no significant differences were observed in the patient background, respiratory function, serum markers associated with AE-ILD, P/F ratio at the onset of AE-ILD, treatment for baseline ILD, treatment for AE-ILD (including nintedanib treatment), and survival.

**Table 3:** The risk factors associated with AE-ILD related to early death (within 90 days) in the univariate analysis.

	Long survival (90 ≥ days) n = 19	Early death (< 90 days) n = 14	Odds ratio	*p*
Age	77.42 ± 9.10	73.85 ± 8.04	1.540	0.214
Male (%)	16 (84.21%)	10 (71.43%)	0.788	0.374
Smoking history (%)	14 (73.68%)	10 (71.43%)	0.021	0.885
IPF (%)	14 (73.68%)	10 (71.43%)	0.021	0.885
UIP + probable UIP on HRCT (%)	18 (94.74%)	12 (85.71%)	0.794	0.372
FVC (%)	68.10 ± 19.02	78.57 ± 20.23	1.098	0.294
DLco (%)	43.02 ± 18.85	30.89 ± 11.31	1.211	0.271
P/F ratio	194.15 ± 83.21	272.76 ± 104.89	4.618	0.031
WBC	11,420.20 ± 4118.90	10,958 ± 2460.40	0.033	0.855
CRP	7.83 ± 6.81	11.68 ± 7.78	1.791	0.180
D-dimer	9.69 ± 14.05	4.69 ± 5.28	0.935	0.333
KL-6	1235.68 ± 598.00	1504.17 ± 1026.00	0.105	0.745
SP-D	537.76 ± 425.11	424.80 ± 279.52	0.331	0.564
LDH	356.73 ± 84.73	365.66 ± 117.44	0.004	0.983
IVCY (%)	9 (47.37%)	9 (64.29%)	0.930	0.334
Immunosuppressant (%)	6 (31.57%)	8 (57.14%)	2.157	0.142
Additional nintedanib administration (%)	9 (47.37%)	2 (14.29%)	3.970	0.046

*AE-ILD* acute exacerbation of interstitial lung disease, *ILD* interstitial lung disease, *OR* odds ratio, *IPF* idiopathic pulmonary fibrosis, *UIP* usual interstitial pneumonia, *HRCT* high resolution computed tomography, *FVC* forced vital capacity, *DLco* diffusing capacity for carbon monoxide, *P/F ratio* PaO_2_/FiO_2_ ratio, *WBC* white blood cell, *CRP* c-related peptide, *LDH* lactate dehydrogenase, *KL-6* Krebs von den Lungen-6, *SP-D* surfactant protein-D, *IVCY* intravenous cyclophosphamide.

**Table 4:** The risk factors associated with AE-ILD related to early death (within 90 days) by multivariate analysis.

	OR	95% CI	*p*
P/F ratio	0.989	0.974–0.997	0.009
Nintedanib	0.107	0.009–0.696	0.016

*OR* odds ratio, *95% CI* 95% confidence interval, *P/F ratio* PaO_2_/FiO_2_ ratio.

## Discussion

Our main findings were as follows: (i) the cumulative mortality rate was significantly lower in the N group than that in the No-N group at 90 days from the onset of AE-ILD according to the generalized Wilcoxon test; (ii) the survival time tended to be longer in the N group than that in the No-N group; (iii) the time to the subsequent AE-ILD was significantly longer in the N group than that in the No-N group; (iv) the incidence of nintedanib-induced gastrointestinal adverse effects, including diarrhea or nausea, was approximately 18% for both; and (v) nintedanib treatment and the P/F ratio were independent prognostic factors after the development of AE-ILD.

In Japan, corticosteroids are typically used for the treatment of AE-ILD^27^. If corticosteroids are ineffective, additional treatment with immunosuppressants is often performed before the EXAFIP result is published^12^. However, initiation of immunosuppressant therapy is difficult in patients with other organ failure, particularly hepatic, renal, or heart failure. Thus, several patients in the N group received nintedanib instead of immunosuppressants. The frequency of receiving IVCY and immunosuppressants was significantly higher in the No-N group than that in the N group. In the EXAFIP results^12^, survival was statistically poorer in the IPF patients who received IVCY than that in those who did not receive IVCY. Although more than half of the patients who received IVCY were diagnosed with non-IPF, including CTD-ILD, IVCY administration may have had an association with survival in our study.

Diffuse alveolar hemorrhage was reported to be one of the pathogeneses of AE-ILD^32,33^. Suppression of the VEGF receptor, which is inhibited by nintedanib, may inhibit vascular endothelial cell regeneration and promote coagulation, resulting in hemorrhage, particularly in cancer^34,35^. Therefore, we considered that nintedanib should not be initiated immediately after the development of AE-ILD. In general, we initiate nintedanib 3 days after the development of AE-ILD at our hospital.

The results of the incidence of subsequent AE-ILD and the time to subsequent AE-ILD in this study were similar to the findings of the incidence of AE-ILD in the Japanese INPULSIS cohort^8^ and the time to first AE-IPF or AE-PF-ILD in the INPULSIS and INBUILD trials^15,17,18^. Previous studies reported that nintedanib prevents or delays the development of AE-ILD. In our study, nintedanib prevented or delayed the relapse of AE-ILD in patients who developed AE-ILD. The prognosis after relapse of AE-ILD is slightly poor. Thus, additional administration of nintedanib may improve the prognosis of patients with ILD.

In this study, multivariate analysis revealed that the P/F ratio and nintedanib treatment after the development of AE-ILD were both independent risk factors associated with early death within 90 days. The odds ratio of nintedanib treatment was lower than that of the P/F ratio. In contrast, the odds ratio of the P/F ratio was close to 1; therefore, nintedanib treatment is considered to be a more important factor associated with early death after the development of AE-ILD compared with a low P/F ratio.

The incidence of nintedanib-related gastrointestinal adverse effects, particularly diarrhea and nausea/anorexia, was low in this study. All patients who developed AE-IPF received steroids. We had recently reported that the incidence of nintedanib-related gastrointestinal adverse effects was significantly lower in patients who received both nintedanib and steroids than that in those who received nintedanib alone, as in this study. Additional steroid treatment may inhibit the development of nintedanib-related gastrointestinal adverse effects^36^. However, no data are available to clarify whether steroid treatment inhibits or prevents nintedanib-related gastrointestinal adverse effects.

We excluded patients who died within 35 days of the development of AE-ILD. Even among patients receiving nintedanib therapy, the effects of the therapy cannot be assessed in patients who have received the drug for a short duration^37,38^. Several patients who died within 35 days underwent intubation and artificial respiratory therapy. These patients could not receive oral medications, including nintedanib. If patients who died early after the development of AE-ILD, particularly within 35 days, are included in the No-N group, the survival in the No-N group may worsen compared with that in the N group. The No-N group may be considered more likely to include patients with poorer prognoses; thus, patients with early death were excluded. In our study, the mortality was remarkably low compared with that reported in previous studies, particularly in the No-N group. Although there was a possibility of differences in the patient background, the P/F ratio, baseline pulmonary function, and other clinical features did not differ between the groups. Moreover, we excluded patients with any advanced cancers. A few of these patients received chemotherapy; thus, patients may develop AE-ILD triggered by chemotherapy. General condition, including PS, was poor in all patients who had advanced cancer before the development of AE-ILD. Thus, we hypothesized that the prognosis would be poorer in patients with advanced cancer than that in those without the development of AE-ILD. Therefore, we excluded patients with advanced cancer.

This study had several limitations. It was a retrospective study with a small sample size. Therefore, the baseline patient characteristics may have been more heterogeneous than those in a prospective study. The small number of patients limited the power of the multivariate analysis that evaluated the risk factors for survival after the development of AE-ILD. We confirmed that there was no significant difference in the baseline patient characteristics, including baseline pulmonary function, between the two groups. There were no statistically significant differences in the HRCT pattern. Although the levels of LDH tended to be higher in the No-N group than those in the N group at the onset of AE-ILD, there was no significant difference in the P/F ratio and serum SP-D level between the two groups. Therefore, we considered that there was no large difference in the patient background between the two groups. Moreover, the small number of patients in the N group may have introduced a serious statistical bias. Although all patients in the N group met the IPF or PF-ILD criteria, there were a few patients in the No-N group who did not meet the IPF or PF-ILD criteria. This difference may have led to the introduction of selection bias. The diagnosis of baseline ILD was heterogeneous. All patients received other treatments including corticosteroids. Although there were no significant differences in the previous treatment for baseline ILD, previous treatments may have influenced survival or the time to subsequent AE-ILD. We confirmed that all patients had a diffuse alveolar damage (DAD) pattern including ground glass opacity with traction bronchiectasis on HRCT. However, no patients were pathologically diagnosed with DAD. Therefore, the diagnosis of DAD may be uncertain. We evaluated the time to subsequent AE-ILD for the surviving patients only. In this analysis, many patients died from courses other than AE-ILD. These deceased patients were censored on the day of death. Therefore, this evaluation may have led to the introduction of selection bias due to the high frequency of censored cases. Additionally, the evaluation of performance status may be one of the indexes for the analysis of the general condition. However, assessing performance status (PS) in the evaluation of the patients’ general condition is not common practice in ILD yet. PS was not documented in the medical record in most cases; thus, we could not assess the difference in general condition between the two groups.

In conclusion, initiating additional nintedanib treatment after the development of AE-ILD may yield better clinical outcomes without severe adverse effects and may be a treatment option for AE-ILD. The publication of further retrospective data similar to that of this study would facilitate conducting future prospective studies to evaluate the efficacy and toxicity of nintedanib for the treatment of patients who developed acute exacerbation to confirm this conclusion.

## Methods

### Patient selection and disease diagnosis

We retrospectively collected data from 85 patients who developed AE-ILD between April 2014 and January 2022. Eleven patients who received nintedanib prior to the development of AE-ILD were excluded from the study as we aimed to evaluate the effect of “additional” nintedanib administration after the development of AE-ILD. Two patients who received nintedanib 35 days after the development of AE-ILD were also excluded. In addition, 28 patients who died within 35 days of developing AE-ILD and 11 patients with active cancer (advanced lung, pharyngeal, and breast cancers) were excluded from the analysis of additional nintedanib treatment. None of the patients who received nintedanib died within 35 days or were complicated with advanced cancer. The remaining patients were divided into those who received additional nintedanib treatment and those who did not. The efficacy and safety of nintedanib treatment were compared between these two groups (Fig. 1).

### AE-ILD definition

In this study, baseline ILD included IPF, idiopathic NSIP, fibrotic HP, and CTD-ILD. The AE-ILD (AE-IPF) criteria were established for patients with IPF. However, AE-ILD associated with progressive ILD other than IPF demonstrated a poor prognosis similar to that of AE-IPF^6^. Thus, we collected the patients with not only IPF but other than IPF. Based on the 2016 American Thoracic Society (ATS) criteria of AE-IPF^1^, AE-ILD was defined as: concurrent or previous baseline diagnosis of ILD; acute progression or development of any symptoms associated with AE-ILD, including cough and dyspnoea within a month; and new bilateral consolidation/ground glass opacity with/without traction bronchiectasis superimposed on baseline findings associated with ILD on high-resolution computed tomography (HRCT). Patients with cardiac/renal failure or those with bacterial pneumonia superimposed on pre-existing ILD were excluded. Two pulmonologists (M.K. and Y.O.) reviewed the HRCT findings prior to the development of AE-ILD based on the ATS/European Respiratory Society/Japan Respiratory Society/Association Latinoamericana del Torax IPF guideline and classified the patterns into four groups^30^. In addition, they also evaluated the HRCT pattern at the onset of AE-ILD based on the previous report by Akira et al.^39^ and confirmed that all included patients had ground glass opacity with traction bronchiectasis as diffuse alveolar damage pattern. Patients who were diagnosed with AE-ILD by the attending physician from the medical record were also included. Subsequently, we confirmed whether the patient’s condition and examination results matched the criteria of AE-ILD. The patients who did not meet the criteria were excluded from our research from the beginning.

### Evaluation

We compared the baseline patient characteristics, including age, sex, smoking history, pulmonary function, diagnosis of ILD (IPF or not IPF), prior treatment for ILD, other treatments for AE-ILD (including steroids and immunosuppressants), ratio of arterial oxygen partial pressure to fractional inspired oxygen (P/F ratio) at the onset of AE-ILD, and serum markers associated with AE-ILD, between the groups. We evaluated the efficacy of nintedanib by assessing the survival time from the development of AE-ILD, time to the subsequent AE-ILD, and cumulative mortality rate at 90 days from the development of AE-ILD.

We also evaluated the risk factors associated with early death due to AE-ILD by performing univariate and multivariate analyses. We performed multivariate analysis using the factors with statistically significant differences in the univariate analysis. In particular, we verified whether nintedanib treatment was a significant independent factor associated with early death. In this study, early death was defined as death within 90 days of the development of AE-ILD, as reported previously^40^. The study protocol was approved by the Juntendo University Ethical Committee and registered under number E21-0332. All methods were performed in accordance with the Declaration of Helsinki and the relevant guidelines and regulations by including a statement. Research Ethics Committee, Faculty of Medicine, Juntendo University waived the requirement for informed consent due to the retrospective nature of the study.

### Statistical analysis

We used the chi-square test, Fisher’s exact test, or Wilcoxon two-sample test to compare patient characteristics between the groups. Parametric and non-parametric data were compared using Student’s t-test and the Mann–Whitney U test, respectively. Survival time and time to subsequent AE-ILD were plotted using the Kaplan–Meier method. Differences in the survival and subsequent AE-ILD were analyzed using the log-rank test and generalized Wilcoxon test. Cox proportional hazard analysis was used to calculate the hazard ratios (HRs), and univariate and multivariate logistic regression analyses were used to determine the risk factors for early death related to AE-ILD. Statistical significance was set at p < 0.05. All statistical analyses were performed using SPSS version 26.0 for Windows (Chicago, IL, USA).

## Data Availability

The datasets generated during and/or analyzed during the current study are available from the corresponding author upon reasonable request.
